# Suspected Endomyocardial Fibrosis With Extensive Left Ventricular Thrombus: A Diagnostic Dilemma

**DOI:** 10.7759/cureus.119032

**Published:** 2026-11-02

**Authors:** Suria Mano Geran, Safiyyah Burhani, Shalu Sharma, Aye T Hline

**Affiliations:** 1 Cardiology, Queen's Hospital, Romford, GBR; 2 Stroke Medicine, The Dudley Group NHS Foundation Trust, Dudley, GBR

**Keywords:** cardiology, endomyocardial fibrosis, eosinophils, heart failure, infiltrative cardiomyopathy, ventricular thrombus

## Abstract

We report a case of a 64-year-old man with biventricular dysfunction and a large left ventricular (LV) thrombus in whom endomyocardial fibrosis (EMF) was suspected. Despite extensive evaluation for eosinophilic, autoimmune, infective, and haematological causes, no secondary aetiology was identified. He was managed with guideline-directed heart failure therapy and anticoagulation. His condition progressively deteriorated, and he died within a year, highlighting the diagnostic challenge of suspected EMF when characteristic imaging findings coexist with atypical features and definitive histological confirmation cannot be obtained, as well as the therapeutic challenges of this rare cardiomyopathy.

## Introduction

Endomyocardial fibrosis (EMF) is a rare restrictive cardiomyopathy characterised by fibrotic involvement of the ventricular endocardium, typically resulting in ventricular apical obliteration, restrictive physiology, atrioventricular valve dysfunction, and intracardiac thrombus. Although predominantly reported in tropical and subtropical regions, EMF may also occur outside endemic areas. The underlying aetiology remains incompletely understood, with eosinophilic cardiac injury and hypereosinophilic syndromes implicated in a subset of cases [1].

Eosinophilic cardiac disease and EMF may exist along a spectrum of endomyocardial injury, with eosinophilic inflammation capable of progressing to thrombosis and EMF. However, distinguishing eosinophilic cardiomyopathy (ECM) from established EMF may be challenging, particularly when peripheral eosinophilia or a clearly identifiable secondary cause is absent. Multimodality imaging, particularly cardiac magnetic resonance (CMR), plays an important role in identifying characteristic endomyocardial abnormalities and thrombus, although definitive diagnosis may remain difficult in atypical cases. We report a case of suspected EMF presenting with biventricular dysfunction and a large ventricular thrombus in a non-endemic setting, underscoring the diagnostic complexity and management challenges [1-4].

## Case presentation

A 64-year-old man was referred to the heart failure clinic following a prolonged period of progressive breathlessness and was subsequently found to have biventricular systolic dysfunction and a large left ventricular (LV) thrombus. Approximately eight months earlier, while visiting family in Pakistan, he had suffered an ischaemic stroke presumptively attributed to embolisation from the LV thrombus. He made a good neurological recovery with minimal residual weakness but did not undergo cardiac assessment until considerably later because of continued travel. Once he returned home, he was referred for an echocardiogram, which identified the LV thrombus and biventricular failure, and he was subsequently referred to the cardiology team.

His medical history included hypertension, subclinical hypothyroidism, and pre-diabetes. He had previously been active and travelled frequently for business. He reported no chest pain, syncope, palpitations, orthopnoea, paroxysmal nocturnal dyspnoea, or peripheral oedema. There were no constitutional, rheumatological, dermatological, or gastrointestinal symptoms. There was no family history of cardiac or thromboembolic disease. He was a lifelong non-smoker, did not consume alcohol, and had no known drug allergies.

On examination, he was clinically stable without signs of congestion. His heart rate was 60 beats/min, and blood pressure was 143/80 mmHg. Electrocardiography demonstrated sinus rhythm with voltage criteria for LV hypertrophy and secondary repolarisation changes.

Initial transthoracic echocardiography (TTE) demonstrated severely impaired LV systolic function, with a visually estimated ejection fraction of approximately 30%, and a possible restrictive diastolic filling pattern (E/A ratio 2.3). A large echodense structure measuring 4.8 × 3.8 cm in the mid-to-apical left ventricle was highly suggestive of an apical thrombus, as seen in Figures 1a-1b. Right ventricular dimensions suggested dilatation, with impaired systolic function evidenced by reduced longitudinal function (TAPSE 1.4 cm) and visually impaired radial function.

**Figure 1:**
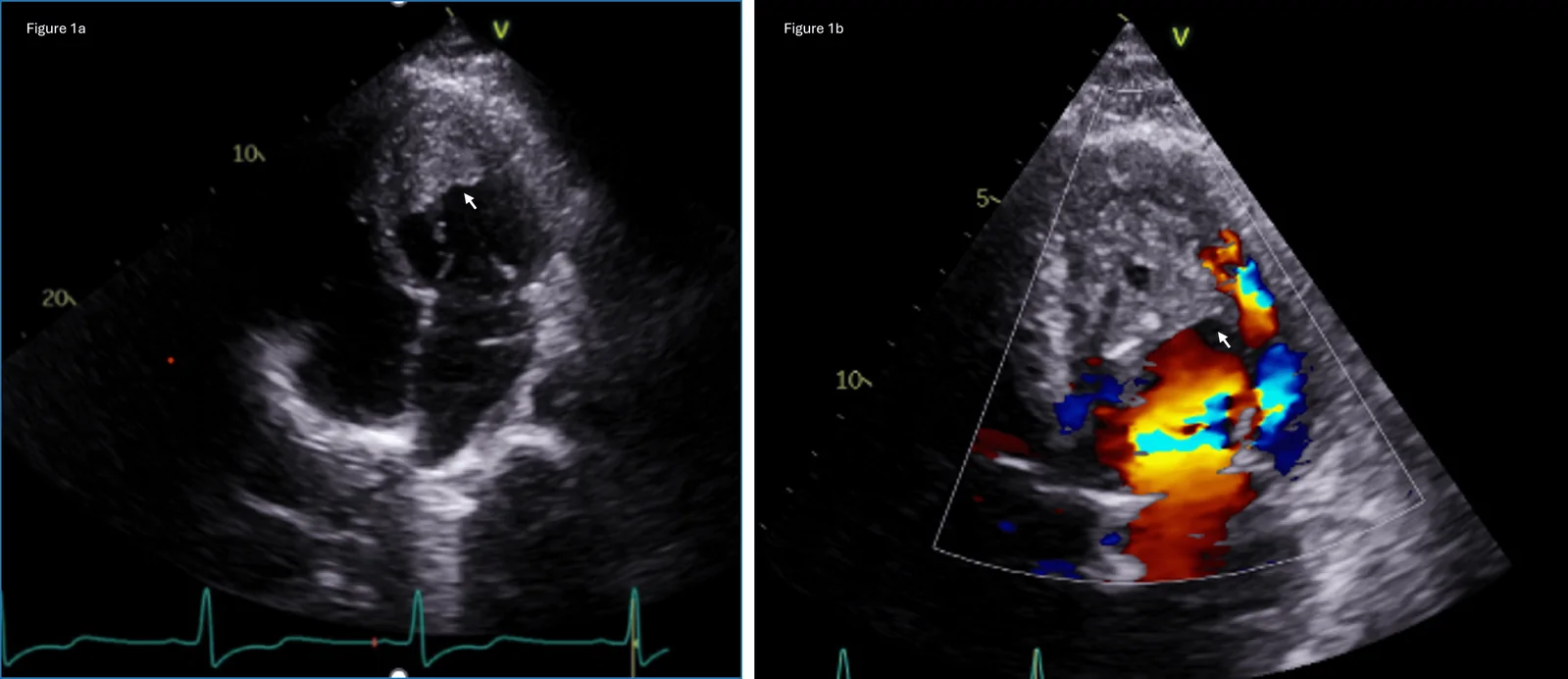
a) TTE apical four-chamber view. b) TTE apical four-chamber view with colour Doppler (the arrow indicates the apical thrombus). TTE: transthoracic echocardiography

Laboratory investigations revealed persistent mild eosinophilia, with an average eosinophil count of 0.9 × 10⁹/L, and a mildly elevated C-reactive protein level of 9 mg/L. Other routine haematological, biochemical, and infectious investigations were unremarkable (Table 1).

**Table 1:** Laboratory investigations in detail.

Test ​	Result ​	Reference Range	Comments
Eosinophil count ​	0.9 x 10⁹/L	0.0-0.5 x 10⁹/L	Mildly elevated
C-Reactive Protein (CRP)	9 mg/L	<5 mg/L	Mildly elevated
ESR ​	2	1-10	Normal
White Cell Count (WCC)	7	3.8-11.0 × 10⁹/L	Normal ​
Haemoglobin (Hb) ​	125	133-173 g/L	Mildly low
Platelet Count (PLT)	292	-	-​
ALT	120	30-130 U/L	Normal
ALP	120	30-130 U/L	Normal
Bilirubin	6	1-21 umol/L	Normal
Albumin	43 ​	35-50 g/L	Normal
Urea	10.4	2.5-7.9 mmol/L	Elevated
Creatinine	122	59-104 umol/L	Elevated
Blood Film	No significant abnormality	-	Normal
Urine Dipstick ​	Negative for blood/protein	-	Normal
Lactate Dehydrogenase (LDH)	181	<100 IU/L	Elevated
Creatine Kinase (CK)	21	20-200 U/L	Low Normal
ANA	Negative	<1 U/mL	Normal
DsDNA	Negative	<1 U/mL	Normal
C3	0.87	0.90-1.80 g/L	Mildly low
C4	0.11	0.10-0.40 g/L	Normal
ACE	36	15-70 U/L ​	Normal
Mast Cell Tryptase	11 µg/L	< 15 µg/L	Within normal limits
Viral Serologies: Toxoplasma, Leptospira, Strongyloides, *Helicobacter pylori* antigen, stool ova, cysts, and parasites (OCP), Toxocara, enterovirus IgM, and Trichinella	Negative for acute infection	-	Normal
Myeloproliferative Panel	Negative	-	-
Serum Electrophoresis	IgG K monoclonal band with IP	-	-
Monoclonal band 1	8	0-0.01 g/dL	Elevated
Serum IgG	11.03	5.5-16.5 g/L	Normal
Serum IgA	0.83	0.80-4.00 g/L	Normal
Serum IgM	0.28	0.50-2.00 g/L	Low
Serum kappa light chain	264.83	3.30-19.4 mg/L​	Elevated
Serum lambda light chain	13.38	5.71-26.3 mg/L	Normal
Kappa:lambda ratio	19.79	0.26-1.65	Elevated

Investigations 

The patient was commenced on guideline-directed medical therapy for heart failure with sacubitril/valsartan, bisoprolol, spironolactone, and dapagliflozin. Anticoagulation with warfarin was initiated following bridging with low-molecular-weight heparin for treatment of the LV thrombus, with a target INR of 2-3. Table 2 shows the most recent INR results for the patient.

**Table 2:** Most recent INR results in anticoagulation clinic.

INR	Result	Reference Range	Comment
Third last INR reading	2.4	2-3	Within range
Second last INR reading	2.1	2-3	Within range
Last INR reading	1.9	2-3	Subtherapeutic

Cardiac magnetic resonance imaging (CMRI) subsequently demonstrated biventricular impairment (LV estimated ejection fraction of approximately 34%) and a large intracavitary mass occupying approximately one-third of the LV cavity. The majority of the mass was avascular and consistent with thrombus, with the overall impression suggesting extensive EMF. LV systolic impairment was predominantly apical, with relatively preserved myocardial function elsewhere despite the extensive thrombus. The overall appearance was considered highly suspicious for EMF. A large unilateral pleural effusion was also present. Figures 2-4 show CMR imaging of the thrombus. 

**Figure 2:**
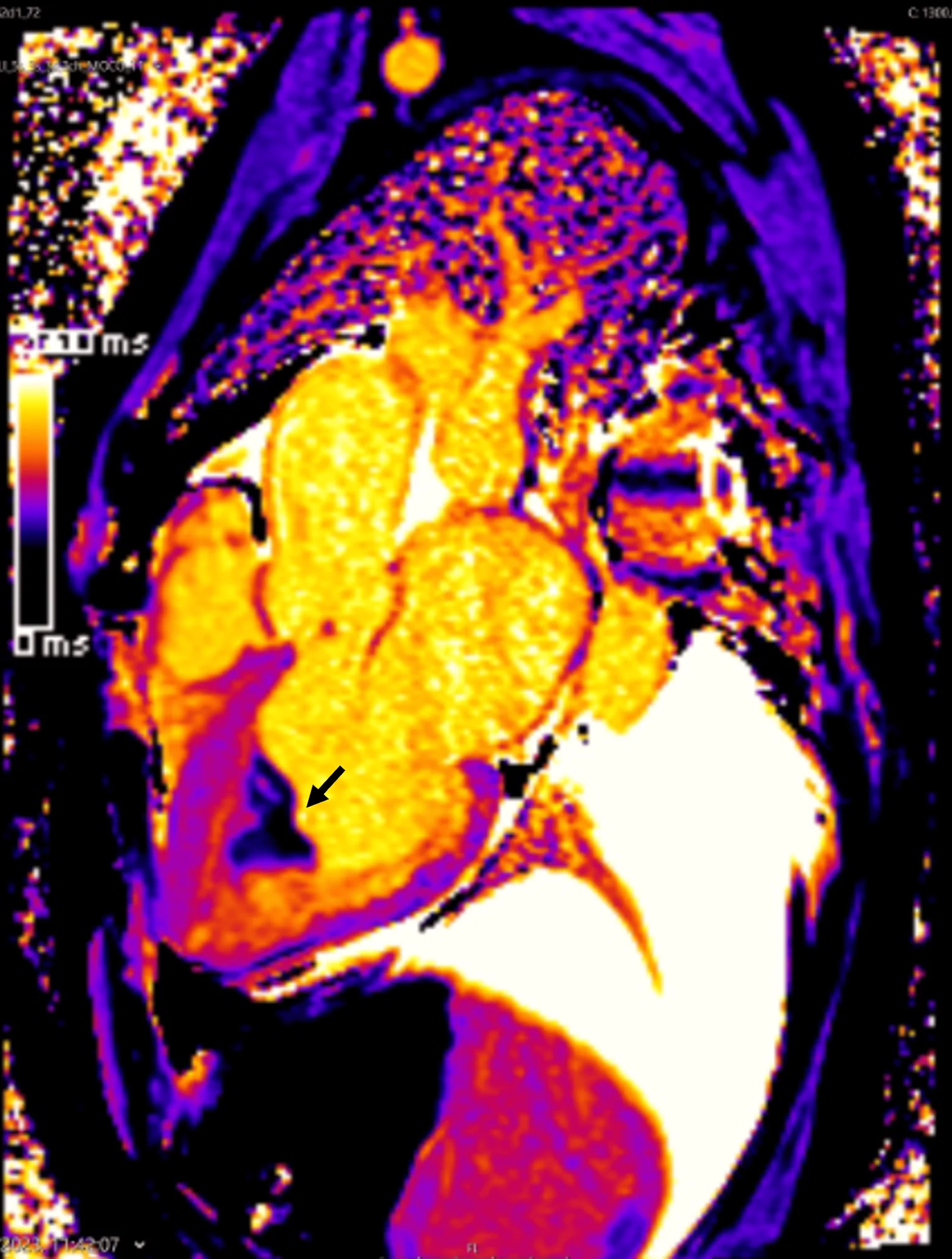
CMRI three-chamber view (ShMOLLI). CMRI two-chamber view with LV thrombus: the characteristic “double-V” sign, representing subendocardial late gadolinium enhancement due to fibrosis with superimposed non-enhancing thrombus, is demonstrated. This imaging appearance has been described as a characteristic feature of endomyocardial fibrosis. The arrow points to the thrombus in the left ventricle. ShMOLLI: Shortened Modified Look-Locker Inversion Recovery; CMRI: Cardiac Magnetic Resonance Imaging; LV: Left Ventricular

**Figure 3:**
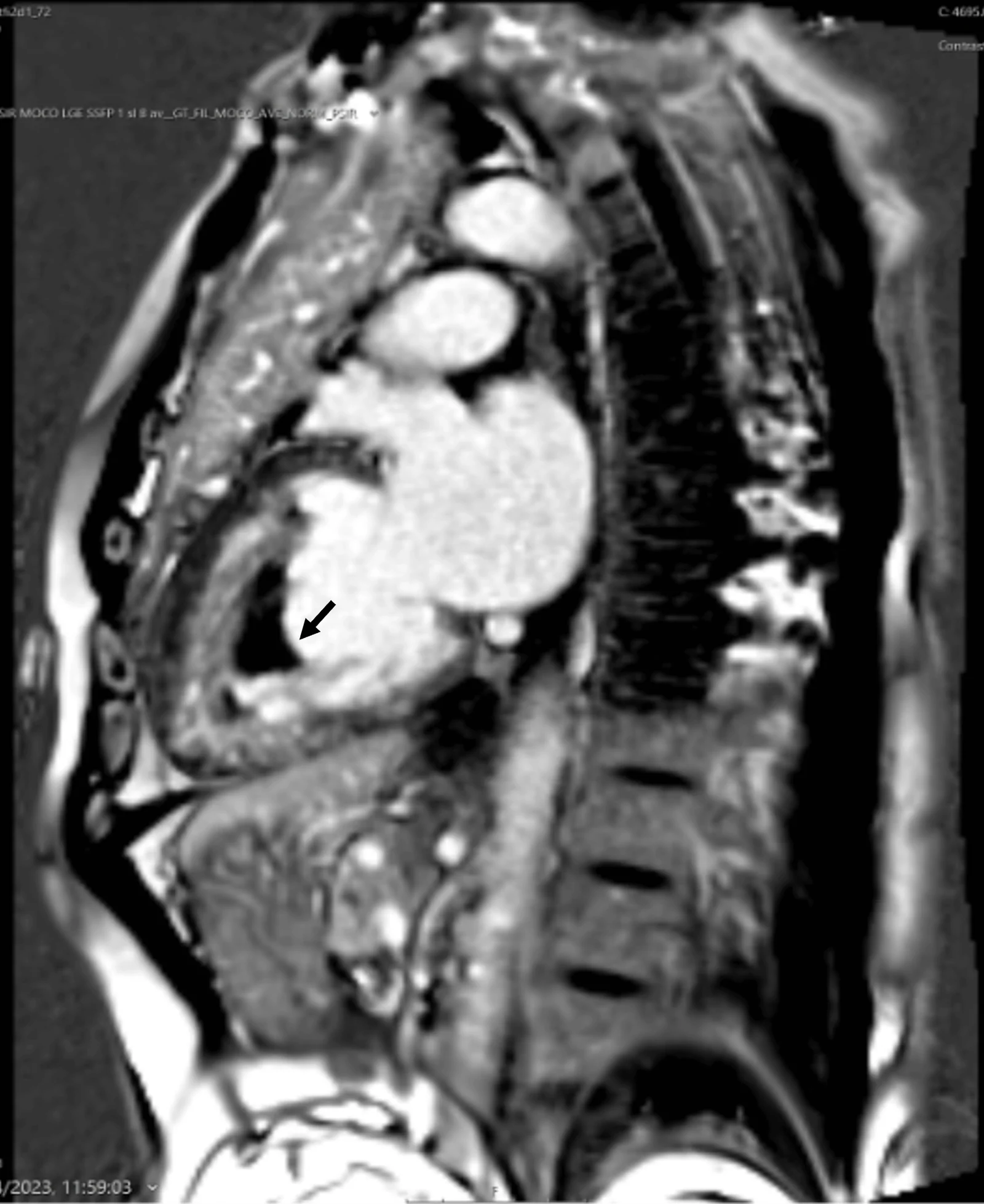
CMRI two-chamber view with LV thrombus. The characteristic “double-V” sign, representing subendocardial late gadolinium enhancement due to fibrosis with superimposed non-enhancing thrombus, is demonstrated. This imaging appearance has been described as a characteristic feature of endomyocardial fibrosis. The arrow points to the thrombus in the left ventricle. CMRI: Cardiac Magnetic Resonance Imaging; LV: Left Ventricular

**Figure 4:**
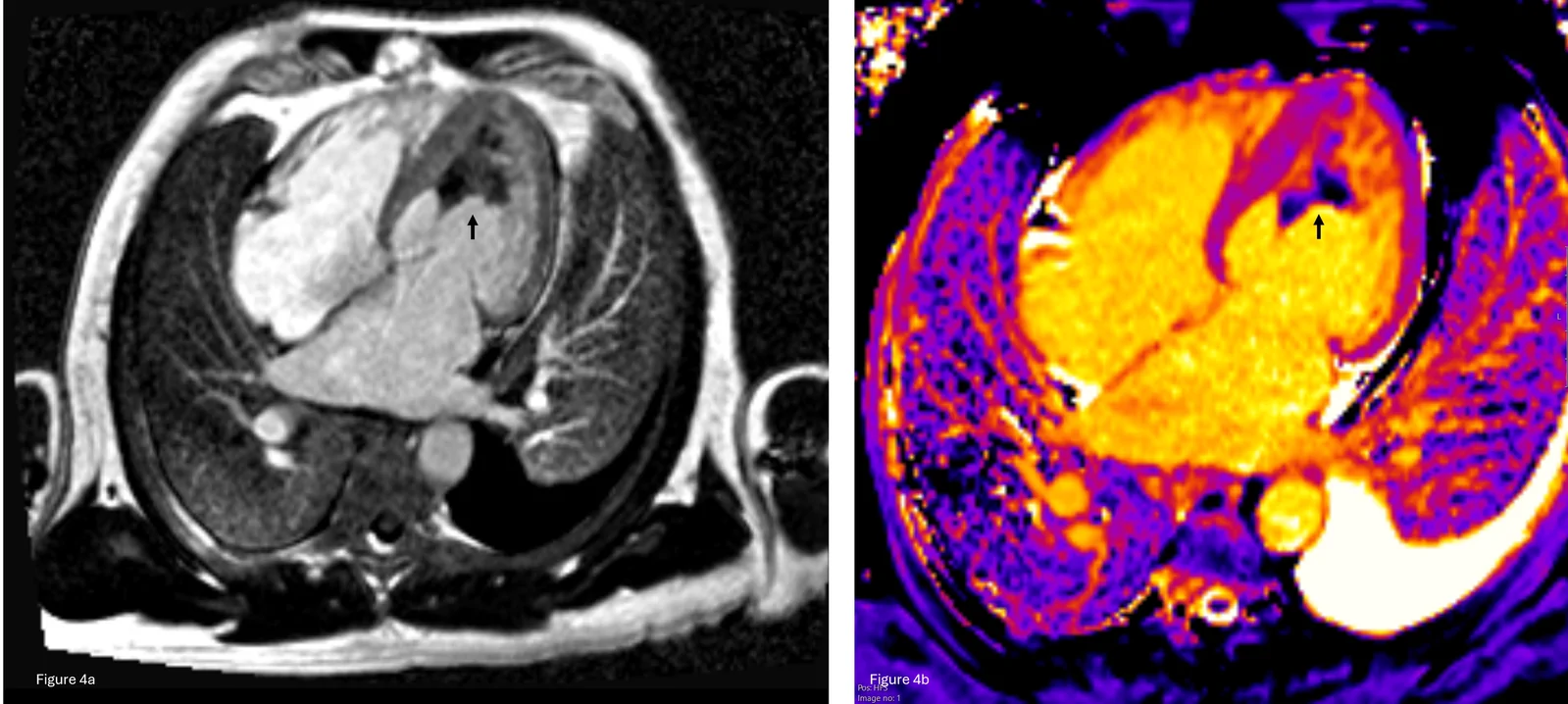
a) CMRI four-chamber view of the LV thrombus; b) CMRI four-chamber view of the LV thrombus with ShMOLLI. The arrow highlights the apical LV thrombus. ShMOLLI: Shortened Modified Look-Locker Inversion Recovery; CMRI: Cardiac Magnetic Resonance Imaging; LV: Left Ventricular

Given the persistent eosinophilia and the possibility of eosinophilic cardiac disease, an extensive investigation for secondary causes was undertaken. Connective tissue, vasculitic, parasitic, and viral investigations were negative, and a myeloproliferative screen was also unremarkable. He was found to have an IgG-kappa paraproteinaemia with an abnormal free light-chain ratio. His serum free light-chain ratio was high at 19.79, and a bone marrow biopsy was recommended. However, the bone marrow biopsy was deferred as the haematology team were concerned about interrupting warfarin therapy in the presence of a persistent LV thrombus. He had mild anaemia and some mild renal dysfunction but no hypercalcaemia. MRI of the spine and pelvis, alongside a skeletal survey, was therefore arranged to assess for lytic bone lesions associated with the abnormal paraprotein. Imaging revealed no evidence of myeloma-related bone disease. In view of these findings and the overall clinical assessment, immediate bone marrow biopsy and treatment were not considered necessary, with ongoing monitoring deemed appropriate.

Positron emission tomography-computed tomography (PET-CT) demonstrated no abnormal uptake. Computed tomography coronary angiography (CTCA) was also performed and demonstrated unobstructed coronary arteries, as seen in Figures 5-7.

**Figure 5:**
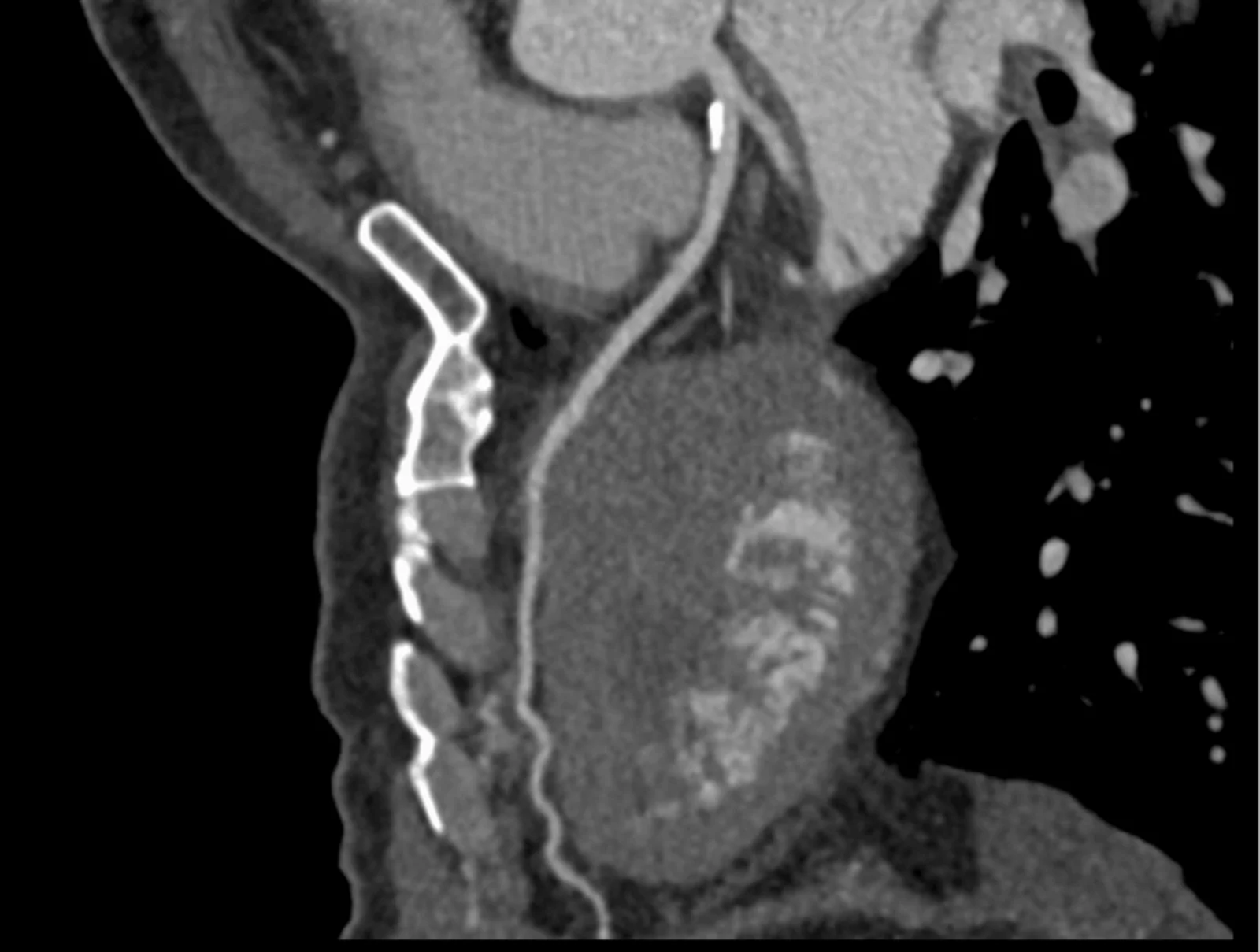
CTCA of left anterior descending artery. CTCA: computed tomography coronary angiography

**Figure 6:**
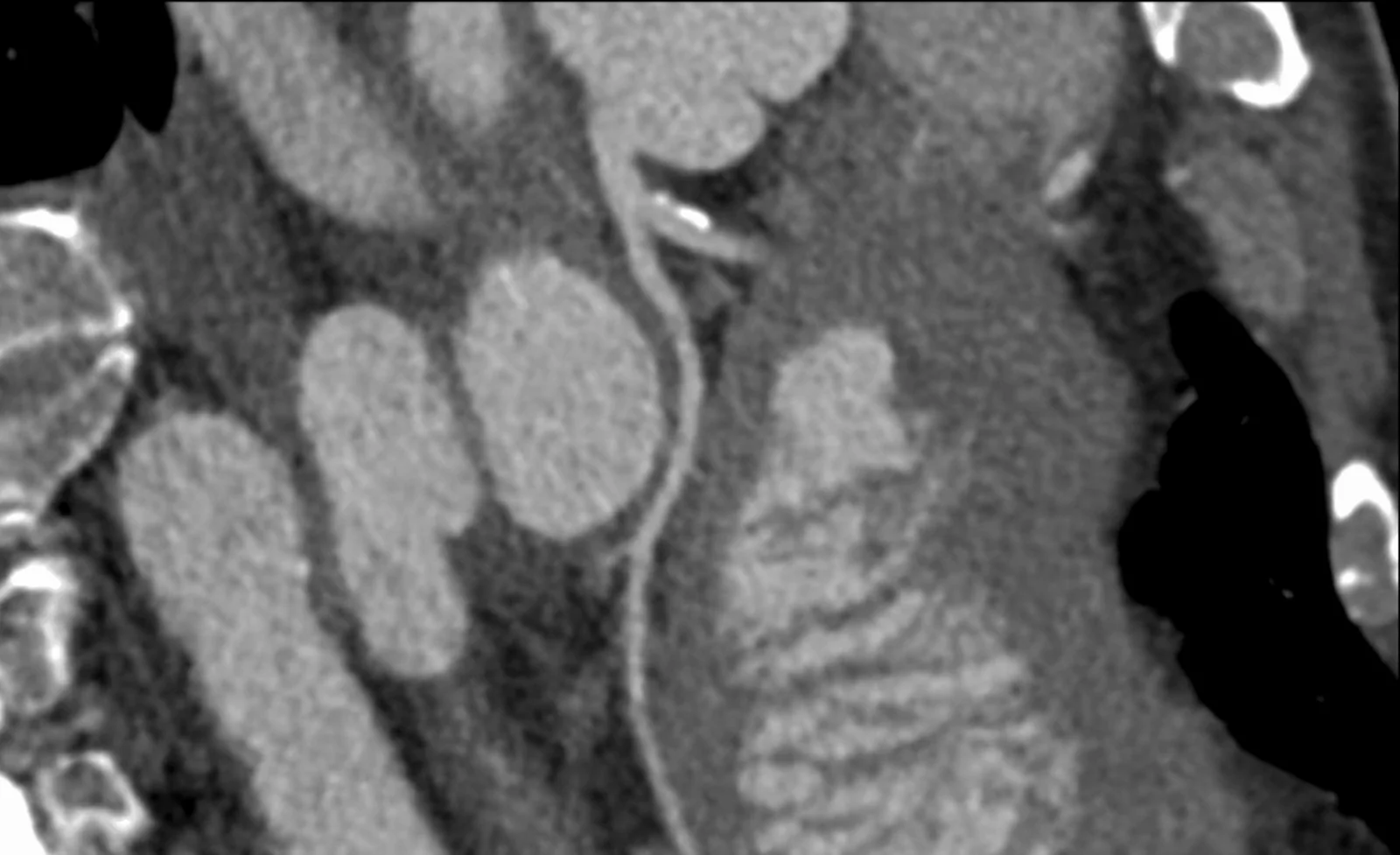
CTCA of left circumflex artery. CTCA: computed tomography coronary angiography

**Figure 7:**
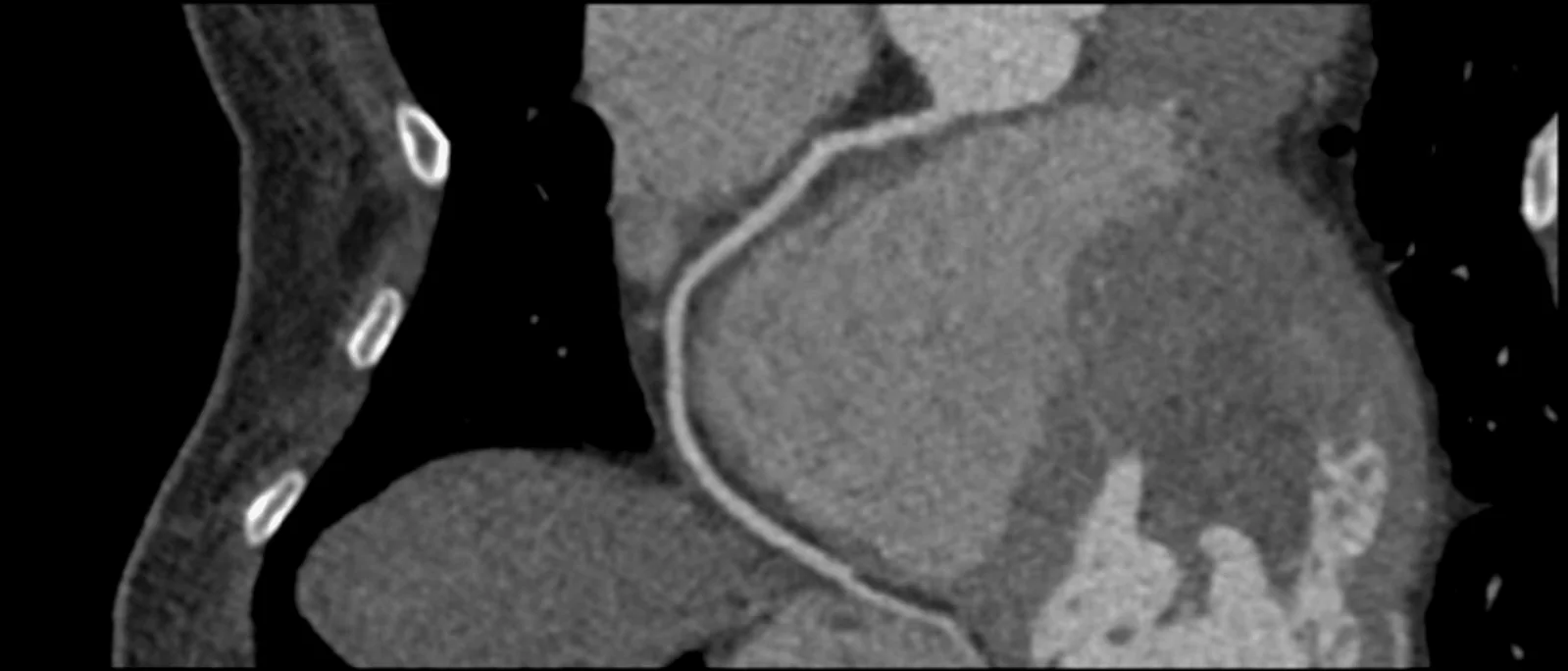
CTCA of right coronary artery. CTCA: computed tomography coronary angiography

Following specialist assessment by the haematology and rheumatology teams, it was felt that there was insufficient evidence to support hypereosinophilic syndrome or systemic autoimmune disease. In the absence of evidence of multi-organ involvement, immunosuppressive therapy was not recommended. Endomyocardial biopsy was discussed to confirm the diagnosis; however, the procedure could not be undertaken as the patient was not able to attend his appointment.

At six-month cardiology follow-up, repeat echocardiography demonstrated persistence of the large LV thrombus with no significant change in systolic function.

Despite ongoing medical therapy and anticoagulation, his clinical condition progressively deteriorated, and the patient passed away a few months later. 

## Discussion

EMF is a rare restrictive cardiomyopathy characterised by progressive fibrosis of the ventricular endocardium. It is predominantly observed in tropical and subtropical regions, where it has been identified as an important cause of heart failure [1]. The exact aetiology of EMF remains uncertain, although several contributing factors have been proposed, including environmental and infectious agents, nutritional deficiencies, genetic and hereditary influences, autoimmune mechanisms, and socioeconomic and lifestyle factors [2].

The pathophysiology of EMF is complex and multifactorial, involving an inflammatory process that may ultimately lead to progressive endocardial fibrosis and cardiac dysfunction [2]. The disease has traditionally been described as progressing through three overlapping stages: an inflammatory or necrotic phase, a thrombotic phase, and a fibrotic phase [2-4]. Patients frequently present once established fibrosis has resulted in restrictive physiology, heart failure, atrioventricular valve dysfunction, or thromboembolic complications. However, there are no specific clinical features that reliably identify the disease during its earlier stages, making early recognition challenging. Although EMF classically produces a restrictive cardiomyopathy, our patient had an atypical presentation with biventricular impairment and an extensive LV thrombus. The subsequent CMR demonstrated LV systolic impairment with left ventricular ejection fraction (LVEF) 34% and a very large intracavitary thrombus.

EMF shares several overlapping features with ECM, particularly in the later stages of disease. Eosinophilic myocardial injury has been proposed as one potential mechanism contributing to EMF, although the relationship between eosinophilia and EMF remains incompletely established [2,3]. Eosinophilic cardiac disease may progress through myocardial injury or necrosis, thrombosis, and ultimately fibrosis, resulting in a phenotype that can overlap substantially with established EMF [3,4]. Distinguishing between active eosinophilic cardiac disease and established EMF is clinically important because treatment strategies differ. In patients with active eosinophilic or inflammatory disease, treatment directed at the underlying process, including immunosuppressive therapy where appropriate, may be indicated, whereas established fibrotic disease is primarily managed by addressing its cardiac manifestations and complications [2,4].

In our patient, persistent mild eosinophilia prompted an extensive investigation for an underlying eosinophilic or systemic disorder. Connective tissue and vasculitic investigations, parasitic and viral screening, and a myeloproliferative screen were negative. An IgG-kappa paraproteinaemia with an abnormal free light-chain ratio was identified; however, there was insufficient evidence of myeloma, and therefore, the patient was kept under haematology for monitoring. Following multidisciplinary assessment involving cardiology, haematology, rheumatology, and infectious diseases, there was insufficient evidence to support hypereosinophilic syndrome or systemic autoimmune disease. In particular, there was no evidence of multi-organ involvement to suggest an active systemic eosinophilic disorder. Immunosuppressive therapy was therefore not pursued. The mild eosinophilia was nevertheless clinically relevant in this case because it prompted investigation of an important potential differential diagnosis rather than being considered diagnostic of ECM.

The diagnosis of EMF in our patient remained presumptive. CMR demonstrated a large intracavitary mass occupying approximately one-third of the LV cavity, the majority of which demonstrated avascular characteristics consistent with thrombus. LV systolic impairment was predominantly apical, with relatively preserved myocardial function elsewhere despite the extensive thrombus. A large unilateral pleural effusion was considered likely to reflect cardiac decompensation.

The overall appearance was considered highly suspicious for EMF. However, the CMR report highlighted diagnostic uncertainty. Thus, although the imaging findings provided substantial support for EMF, this did not establish the diagnosis with certainty.

Multimodality cardiac imaging plays a central role in the assessment of suspected EMF. Echocardiography can demonstrate features such as apical obliteration, endocardial thickening, ventricular cavity reduction, atrioventricular valve dysfunction, and intracardiac thrombus. CMR provides additional structural and tissue-characterisation information and may demonstrate characteristic patterns of endocardial late gadolinium enhancement and apical involvement [2,5,6]. The “double-V” sign, reflecting subendocardial fibrosis with superimposed thrombus, has been described as a characteristic imaging feature of EMF [7]. However, in an atypical presentation such as this, characteristic imaging findings should be interpreted in the context of the clinical picture and alternative diagnoses, particularly when histological confirmation is not available.

An antecedent transient myocardial insult, including myocardial stunning or Takotsubo syndrome, was considered as a potential explanation for the ventricular dysfunction and subsequent thrombus formation. However, the abnormalities persisted on serial imaging over several months without significant recovery, making a transient myocardial stunning syndrome less likely. CTCA demonstrated unobstructed coronary arteries. The persistence of the abnormalities, together with the characteristic endomyocardial appearances on CMR, therefore maintained EMF as the leading suspected diagnosis, although definitive confirmation remained unavailable.

The extensive thrombotic burden was a particularly important feature of this case. The patient had sustained an ischaemic stroke attributed to embolisation from the LV thrombus before his subsequent cardiac assessment, and the large thrombus remained present despite anticoagulation. This suggests that thromboembolic disease was an important manifestation of the underlying cardiac process. The degree of clinical deterioration was notable in the context of the relatively preserved myocardial function outside the apical region. However, the extent to which the thrombus itself contributed directly to the patient's haemodynamic deterioration cannot be established with certainty.

Endomyocardial biopsy was considered to obtain histological confirmation and further investigate potential inflammatory or infiltrative myocardial disease. However, although the patient was referred for the procedure, he did not attend the scheduled appointment, and biopsy was therefore not performed. Consequently, the diagnosis of EMF remained presumptive, based on the characteristic CMRI findings and the overall clinical presentation. This highlights an important diagnostic limitation, as histological confirmation could not be obtained despite comprehensive multimodality imaging and specialist assessment.

The management of established EMF is largely directed towards the consequences of ventricular fibrosis and associated restrictive or systolic dysfunction, as well as complications such as intracardiac thrombosis, systemic embolisation, arrhythmia, and valvular dysfunction [2]. In patients with active eosinophilic disease, treatment of the underlying eosinophilic process may be appropriate; however, there was insufficient evidence in our patient to support such an approach. He was therefore managed with guideline-directed medical therapy for heart failure and anticoagulation for the extensive LV thrombus.

Surgical options for EMF are limited and are generally considered on an individual basis. Surgical endocardectomy has been described in selected patients with advanced disease, particularly where there is severe endocardial involvement, restrictive physiology, or significant atrioventricular valve dysfunction [2,8,9]. Heart transplantation may also be considered in selected patients with advanced refractory disease. In our patient, the extensive unresolved LV thrombus, overall clinical condition, and diagnostic uncertainty limited the applicability of surgical approaches. Despite treatment with guideline-directed heart failure therapy and anticoagulation, the thrombus persisted at six-month follow-up, with no significant change in systolic function. His clinical condition subsequently deteriorated, and he died several months later.

## Conclusions

This case highlights the diagnostic dilemma of suspected EMF in a patient presenting with an extensive left ventricular thrombus, biventricular impairment, and persistent mild eosinophilia. Although CMR demonstrated features highly suspicious for EMF, the diagnosis could not be definitively established because endomyocardial biopsy was considered unsafe in the presence of the large ventricular thrombus. Extensive investigation did not identify an underlying eosinophilic, autoimmune, infective, or haematological disorder, and there was insufficient evidence to support hypereosinophilic syndrome or systemic eosinophilic disease. 

This case emphasises the value of multimodality imaging and multidisciplinary assessment to narrow down the clinical suspicion, in this case, EMF, while highlighting the diagnostic uncertainty that may remain when characteristic imaging findings cannot be corroborated histologically. It also illustrates the importance of distinguishing suspected EMF from active ECM, as identification of an underlying eosinophilic or inflammatory disorder may have important therapeutic implications. 
